# EuPdSn_2_: magnetic structures in view of resonant X-ray Bragg diffraction

**DOI:** 10.1107/S2052520625006134

**Published:** 2025-08-15

**Authors:** Stephen W. Lovesey

**Affiliations:** a ISIS Facility STFC Didcot OxonOX11 0QX UK; b Diamond Light Source, Harwell Science and Innovation Campus, Didcot, OxonOX11 0DE, UK; c Department of Physics, Oxford University, Oxford, OX1 3PU, UK; Politecnico di Milano, Italy

**Keywords:** magnetic, resonant X-ray diffraction, structure factors, magnetic multipoles

## Abstract

The magnetic properties of materials hosting Eu^2+^ (*J* = 7/2, 4*f*^7^) ions have attracted much attention in the science of strongly correlated electrons. In part because crystal electric field effects are impoverished for an *S*-state ion, as with Gd^3+^ intermetallics, and Eu^2+^ substitution in biological and optically active materials is resourceful. The magnetic structure of EuPdSn_2_ is not wholly resolved. Ferromagnetic and antiferromagnetic structures coexist in powder neutron diffraction patterns, and compete in the ground state. Moreover, the specific heat as a function of temperature is enigmatic and indicative of *J* = 5/2. We present symmetry-informed analytic magnetic structure factors for single crystal resonant X-ray Bragg diffraction using Eu atomic resonances that reveal significant potential for the technique. Europium ions use Wyckoff positions that are not centres of inversion symmetry in magnetic space groups inferred from neutron diffraction. In consequence, axial and polar Eu multipoles are compulsory components of both magnetic neutron and resonant X-ray Bragg diffraction patterns. The proposed antiferromagnetic phase of EuPdSn_2_ supports anapoles (magnetic polar dipoles) already observed in magnetic neutron diffraction patterns presented by Gd-doped SmAl_2_, and several resonant X-ray diffraction patterns.

## Abstract 2

Structure factors informed by magnetic symmetry for resonant X-ray diffraction by EuPdSn_2_ using Eu atomic resonances *E*1–*E*1 and *E*1–*E*2. Axial and polar (Dirac) electronic Eu multipoles are compulsory.

## 1. Introduction

Interpretations of major studies of the magnetic structure of EuPdSn_2_ by neutron diffraction on powder samples indicate the presence of both ferromagnetic (FM) and antiferromagnetic (AF) structures (Martinelli *et al.*, 2023 ▸; Sereni *et al.*, 2025 ▸). The structures coexist below a temperature ≈ 12 K, and compete in the ground state. The efficacy of neutron diffraction for this compound is curtailed by the high-absorption cross-section of natural Eu. Looking ahead, we present symmetry-informed analytic amplitudes for X-ray diffraction by EuPdSn_2_ with the primary energy tuned to an Eu atomic resonance (Ruck *et al.*, 2011 ▸; Anderson *et al.*, 2017 ▸). Bragg diffraction patterns for FM and AF structures are significantly different.

In the theory of resonant X-ray Bragg diffraction used here (Lovesey *et al.*, 2005 ▸; Lovesey & Balcar, 2013 ▸), electronic properties of Eu ions are encapsulated in spherical atomic multipoles of rank *K*. They are properties of the magnetic ground state. Valence states accessed by photo-ejected electrons interact with neighbouring ions when X-rays excite a core resonance. The aspherical rotational symmetry of Eu electronic multipoles matches the symmetry of its Wyckoff position (Neumann principle; Cracknell, 1975 ▸). Absence conditions in Bragg diffraction can be violated by relatively weak spots arising from non-spherical atomic charge (Templeton & Templeton, 1985 ▸). Tuning the energy of X-rays to an atomic resonance has two obvious benefits. First, there is a welcome enhancement of Bragg spot intensities and, second, spots are element specific. There are four scattering amplitudes labelled by photon polarization, two with unrotated and two with rotated states of polarization (Lovesey *et al.*, 2005 ▸; Scagnoli & Lovesey, 2009 ▸; Paolasini, 2014 ▸). Strong Thomson scattering, by spherically symmetric atomic charge densities, is absent in rotated channels of polarization for axial electric dipole–electric dipole (*E*1–*E*1) and electric quadrupole–electric quadrupole (*E*2–*E*2) absorption events. It is, however, allowed in unrotated channels of polarization using *E*1–*E*1 and *E*2–*E*2 events. Thomson scattering is absent in a parity-odd absorption using a polar electric dipole–electric quadrupole (*E*1–*E*2) event, for example. The range of values of the multipole rank *K* is fixed by the triangle rule, with *K* = 0–2, *K* = 1–3 and *K* = 0–4 for *E*1–*E*1, *E*1–*E*2 and *E*2–*E*2 events, respectively.

## 2. Unit-cell structure factor

**Figure 1:**
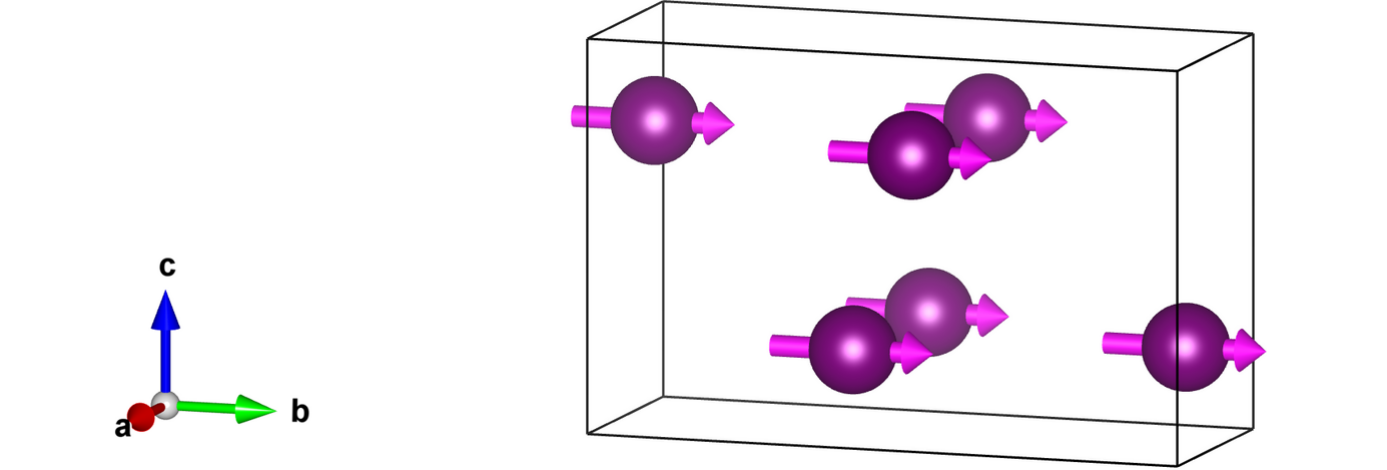
Ferromagnetic (FM) phase *Cm*′cm′ (BNS No. 63.464) of EuPdSn_2_ determined by powder neutron diffraction. The FM phase develops between 13.4 K and ≈ 10 K (Martinelli *et al.*, 2023 ▸).

Both axial and polar Eu multipoles are included in our FM and AF diffraction patterns. In so doing, we comply with an edict whereby anything not forbidden by symmetry is compulsory. It is known in other circles as the ‘totalitarian principle’ of symmetry attributed to Murray Gell-Mann (Milton, 2006 ▸). Non-magnetic monopoles (*K* = 0) present Thomson scattering at space-group allowed reflections. A magnetic monopole possesses the discrete symmetries of a Dirac monopole (Milton, 2006 ▸). The two types of magnetic dipoles (*K* = 1) are atomic moments, featured in Figs. 1 ▸ and 2 ▸, and an anapole (Dirac dipole) depicted in Fig. 3 ▸ (Scagnoli *et al.*, 2011 ▸; Lovesey *et al.*, 2019 ▸). Dirac quadrupoles (*K* = 2) occur in theories of spintronic and multiferroic materials, *e.g.* GdCrO_3_ (Manuel *et al.*, 2025 ▸; Hayami, 2025 ▸).

A universal spherical structure factor of rank *K*
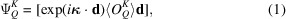
determines the chemical (nuclear) and magnetic Bragg diffraction patterns for a reflection vector **κ** defined by integer Miller indices (*h*, *k*, *l*) (Scagnoli & Lovesey 2009 ▸). The implied sum is over Eu ions in sites **d**. The generic electronic multipole 〈*O*^*K*^_*Q*_〉 possesses (2*K* + 1) projections in the interval −*K* ≤ *Q* ≤ *K*, and its complex conjugate is defined by (−1)^*Q*^〈*O*^*K*^_−*Q*_〉 = 〈*O*^*K*^_*Q*_〉*. Angular brackets denote a time-average, or expectation value, of the enclosed spherical tensor operator. Our phase convention for real and imaginary parts labelled by single and double primes is 〈*O*^*K*^_*Q*_〉 = [〈*O*^*K*^_*Q*_〉′ + *i*〈*O*^*K*^_*Q*_〉′′]. Cartesian dipole moments in a unit cell (ξ, η, ζ) are 〈*O*^1^_ξ_〉 = −√2 〈*O*^1^_+1_〉′, 〈*O*^1^_η_〉 = −√2 〈*O*^1^_+1_〉′′, and 〈*O*^1^_ζ_〉 = 〈*O*^1^_0_〉.

Multipoles engaged by *E*1–*E*1 and *E*2–*E*2 absorption events are parity-even (parity signature σ_π_ = +1) to match the axial spatial symmetry. They are time-even with a time signature σ_θ_ = +1 (time-odd σ_θ_ = −1) for even (odd) rank *K*, *i.e.*σ_θ_ (−1)^*K*^ = +1. Parity-odd (σ_π_ = −1) multipoles match the spatial symmetry of the polar *E*1–*E*2 absorption event. Discrete symmetries σ_θ_σ_π_ = +1 define Dirac multipoles (Milton, 2006 ▸). Lovesey & Balcar (2013 ▸) give a formal derivation of the cited time signatures. Multipoles have been estimated from simulations of the electronic structure and analytic wavefunctions. Ovchinnikova *et al.* (2025 ▸) report studies of several compounds using the FDMNES simulation code (Bunău *et al.*, 2022 ▸), including the iron *K*-edge of iron orthoborate Fe_3_BO_6_ and the uranium *M*_4_ edge of U_2_N_3_. An analytic form of the Cu atomic wavefunction in CuO yields a satisfactory interpretation of observed Dirac multipoles (Scagnoli *et al.*, 2011 ▸; Lovesey & Balcar, 2013 ▸).

The structure factor Ψ^*K*^_*Q*_ is informed of all elements of symmetry in the magnetic space group. In more detail, equation (1) possesses information about the relevant Wyckoff positions available in the Bilbao table MWYCKPOS for the magnetic symmetry of interest [Bilbao Crystallographic server, http://www.cryst.ehu.es, Belov–Neronova–Smirnova (BNS) setting of magnetic space groups]. Site symmetry that might constrain projections *Q* is given in the same table. Wyckoff positions in a unit cell are related by operations listed in the table MGENPOS (Bilbao). Taken together, the two tables provide all information required to evaluate equation (1) and, thereafter, Bragg diffraction patterns.

The photon wavelength λ = (2πħ*c*/*E*) with energy *E*, Planck’s constant ħ, and the velocity of light *c*. For the Laue condition we use λ = (12.40/*E*) (Å) with *E* (keV), to a good approximation. Atomic Eu absorption events of immediate interest in resonant X-ray Bragg diffraction by EuPdSn_2_ include the *K* edge ≈ 48.49 keV, *L*_2_ ≈ 7.62 keV, *L*_3_ ≈ 6.98 keV (2*p* → 5*d*), and *M*_4,5_ ≈ 1.13 keV (3*d* → 4*f*) (Thole *et al.*, 1985 ▸; Ruck *et al.*, 2011 ▸). Unit-cell dimensions for EuPdSn_2_ are *a* ≈ 4.4480 (1) Å, *b* ≈ 11.5420 (1) Å, *c* ≈ 7.4266 (1) Å (Martinelli *et al.*, 2023 ▸). Laue conditions for reflections (*h*, 0, *l*) and (0, *k*, *l*) for the FM and AF magnetic phases, respectively, follow from
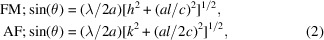
where θ is the Bragg angle. Factors (λ/2*a*) ≈ 1.23 and (λ/2*c*) ≈ 0.74 for *M*_4,5_, and there are no FM Bragg spots. For the Eu *L*_2_ edge (λ/2*a*) ≈ 0.18 and reflections with even *h*, *l* are allowed in the FM diffraction pattern. The *L*_2_ and *L*_3_ edges access 5*d* and 4*f* orbitals with *E*1 and *E*2 transitions, respectively.

In keeping with standard notation, photon polarizations parallel and perpendicular to the plane of scattering are labelled by π and σ, respectively (Lovesey *et al.*, 2005 ▸; Scagnoli & Lovesey, 2009 ▸; Paolasini, 2014 ▸). Diffraction amplitudes labelled (σ′σ) and (π′π) denote scattering with no rotation of the polarization, *e.g.* σ → σ′. The two remaining amplitudes (π′σ) and (σ′π) entail the rotation of polarization. In the theory of resonant X-ray diffraction adopted here intensity of a Bragg spot = |(π′σ)|^2^, for example.

## 3. FM phase

Axial magnetic dipoles allowed in the FM space group *Cm*′*cm*′ (BNS No. 63.464) are depicted in Fig. 1 ▸. Europium ions use Wyckoff positions (4*c*) with *y* ≈ 0.4339 (Martinelli *et al.*, 2023 ▸). The orthorhombic centrosymmetric magnetic crystal class *m*′*mm*′ permits ferromagnetism, a nonlinear magnetoelectric effect, and the piezomagnetic effect. The FM phase develops between 13.4 K and ≈ 10 K (Martinelli *et al.*, 2023 ▸), and the corresponding structure factor is
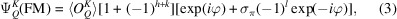
with φ = 2π*k*y and even (*h* + *k*) from the centring condition. Wyckoff position symmetry *m*′2*m*′ does not contain inversion. Rotation symmetry elements demand σ_π_ σ_θ_ (−1)^*Q*^ = +1, and 〈*O*^*K*^_*Q*_〉 = (−1)^*K*^^+*Q*^〈*O*^*K*^_−*Q*_〉 = (−1)^*K*^〈*O*^*K*^_*Q*_〉^*^. Notably, 〈*O*^*K*^_0_〉 is permitted for even *K*.

Parity-even multipoles 〈*T*^*K*^_Q_〉 possess a time signature σ_θ_ = (−1)^*K*^, and it leads to even (*K* + *Q*). Dipoles *K* = 1 possess *Q* = ±1 and are confined to the *ab* plane. Bulk magnetic signals, such as X-ray magnetic circular dichroism (XMCD), are proportional to Ψ^*K*^_*Q*_(FM) with Miller indices *h* = *k* = *l* = 0 (Lovesey *et al.*, 2005 ▸; Anderson *et al.*, 2017 ▸). Equation (3) evaluated with σ_π_ = +1 and σ_θ_ = −1 yields Ψ^1^_+1_(FM) = *i*4〈*T*^1^_+1_〉′′, and bulk ferromagnetism parallel to the *b* axis. Dirac multipoles 〈*G*^*K*^_*Q*_〉 are revealed in the parity-odd *E*1–*E*2 absorption event that requires σ_π_σ_θ_ = +1, which leads to even *Q*, and a magnetic monopole 〈*G*^0^_0_〉.

The fact that 〈*T*^*K*^_0_〉 with even rank is permitted in the FM phase means that Thomson scattering 〈*T*^0^_0_〉 contributes to unrotated diffraction amplitudes (σ′σ) and (π′π) for *E*1–*E*1 and *E*2–*E*2 events (Scagnoli & Lovesey, 2009 ▸). This is not so for the rotated amplitude (π′σ), however. For a reflection vector **κ** = (*h*, 0, *l*) with even *h*, *l* and an *E*1–*E*1 event
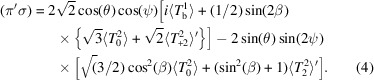
The azimuthal angle ψ measures rotation of the crystal about **κ**, and the orthorhombic *b* axis is normal to the plane of scattering for ψ = 0. The angle β in equation (4) is fixed by cos(β) = *h*/[*h*^2^ + (*al*/*c*)^2^]. Note that (π′σ) is proportional to cos(ψ), and the dipole parallel to the crystal *b* axis is 90° out of phase with contributions from quadrupoles. For (0, 0, 2*n*) Templeton–Templeton scattering (Templeton & Templeton, 1985 ▸) 〈*T*^2^_+2_〉′ survives alongside 〈*T*^1^_b_〉. Dirac multipoles 〈*G*^*K*^_*Q*_〉 do not exist in the paramagnetic phase. They are characterized by σ_π_ σ_θ_ = +1, and even *Q* in the FM phase. An anapole (*K* = 1) as depicted in Fig. 3 ▸ does not contribute to reflections (*h*, 0, *l*) with even *h*, odd *l* using an *E*1–*E*2 event. Quadrupole contributions to (σ′σ) and (π′σ) are
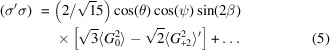

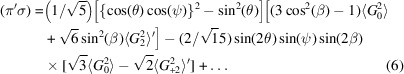
Octupoles (*K* = 3) are omitted here on the grounds of simplicity; they are readily constructed from available universal expressions (Scagnoli & Lovesey 2009 ▸). There is no quadrupole contribution to (σ′σ) for a reflection (0, 0, 2*n*), and (π′σ) reduces to an even function of the azimuthal angle.

## 4. AF phase

**Figure 2:**
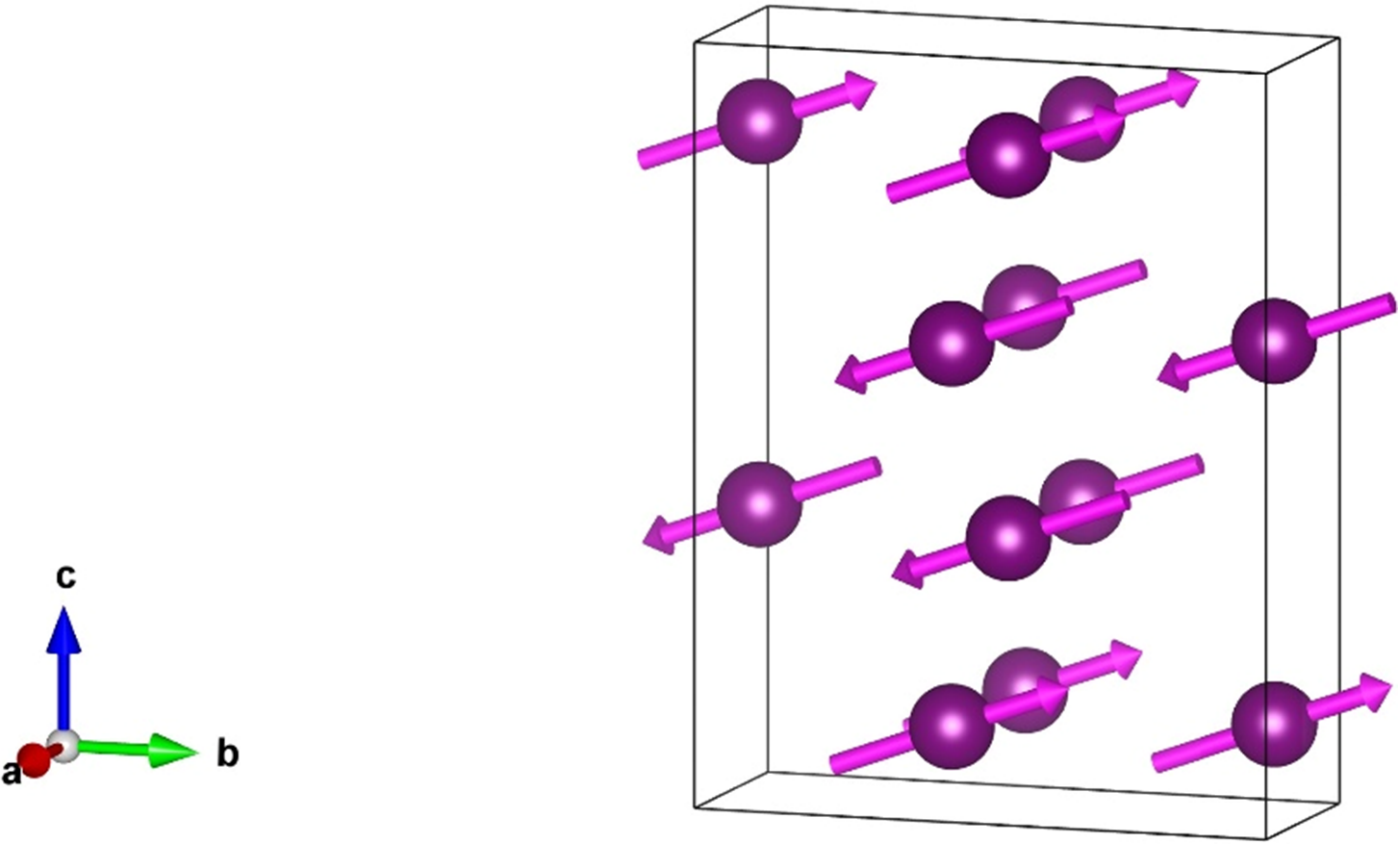
Antiferromagnetic (AF) phase *C*_*c*_2/*c* (BNS No. 15.90) of EuPdSn_2_ is observed below 12.3 K and the transition completes below ≈ 4 K (Martinelli *et al.*, 2023 ▸).

The AF structure C_c_2/*c* (No. 15.90) is depicted in Fig. 2 ▸. Europium ions use Wyckoff positions (8*i*) at (0.5610, 0, 1/8). A basis {(0, −1, 0), (1, 0, 0), (0, 0, 2)} relative to the parent structure defines orthogonal local axes (ξ, η, ζ) for an Eu ion. The monoclinic centrosymmetric structure belongs to the magnetic crystal class 2/*m*1′ for which any kind of magnetoelectric effect is prohibited. It is a grey group that contains all three inversions 1, 1′, 1′. Ferromagnetism and the piezomagnetic effect are forbidden. The AF magnetic phase is observed below 12.3 K and the transition completes below ≈ 4 K (Martinelli *et al.*, 2023 ▸), and the corresponding structure factor is
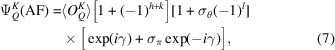
with γ = {π(2*hx* + *l*/4)} and *x* ≈ 0.5610. Magnetic properties are visible for odd *l*, which is a forbidden chemical (nuclear) reflection. A null bulk value of Ψ^*K*^_*Q*_(AF) is correct for antiferromagnetic order. Symmetry of the Wyckoff position (8*i*) does not include inversion, and rotation elements demand 〈*O*^*K*^_*Q*_〉 = {σ_π_ σ_θ_(−1)^*K*^^+ *Q*^ 〈*O*^K^_−*Q*_〉}.

For an *E*1–*E*1 event 〈*T*^*K*^_*Q*_〉 = 〈*T*^*K*^_*Q*_〉*, and 〈*T*^*K*^_0_〉 is permitted for all *K*. Allowed axial dipoles are 〈*T*^1^_0_〉 and 〈*T*^1^_ξ_〉, *i.e.* dipoles are parallel to the orthorhombic *c* and *b* axes. The condition even (*K* + *l*) follows from the *E*1–*E*1 time signature σ_θ_ (−1)^*K*^ = +1, and forbidden reflections with odd *l* are purely magnetic. The *E*1–*E*1 amplitude (σ′σ) = 0, because it does not include multipoles with odd *K* (Scagnoli & Lovesey, 2009 ▸). The remaining amplitudes are purely imaginary with a common factor [*i*4√2cos(π*l*/4)] that is omitted in the results
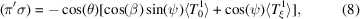

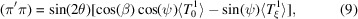
with cos(β) = {(λ*k*)/[2*a*sin(θ)]}. The azimuthal angle ψ measures rotation of the crystal sample about the reflection vector (0, *k*, *l*), and the monoclinic ξ axis is normal to the plane of scattering for ψ = 0.

**Figure 3:**
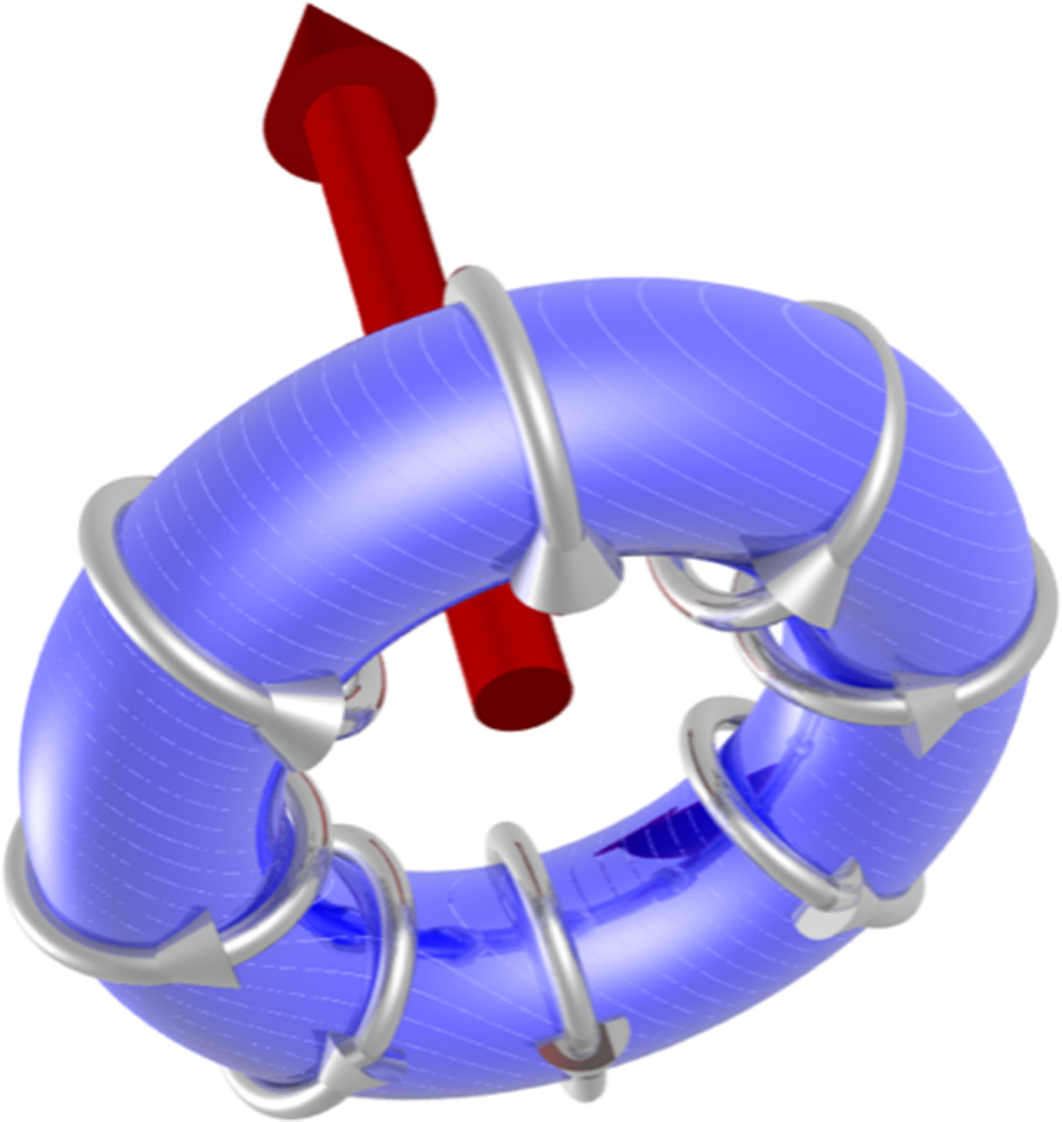
Depiction of an anapole, also known as a toroidal dipole (Scagnoli *et al.*, 2011 ▸).

Unlike an *E*1–*E*1 event, the parity-odd *E*1–*E*2 amplitude in the unrotated channel of polarization can be different from zero. It reveals the anapole depicted in Fig. 3 ▸ parallel to the orthorhombic *a* axis 〈*G*^1^_η_〉. Reflections (0, *k*, *l*) require even *k* and odd *l*. At the level of the anapole and quadrupoles the amplitude is
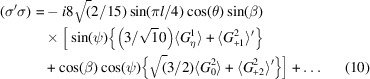
Notably, sin(β) ∝ *l* and it is different from zero for all considered reflections.

## 5. Conclusions

In summary, we present exact analytic amplitudes for resonant X-ray Bragg diffraction from EuPdSn_2_ using an Eu atomic absorption event. A major study of a powder sample of the compound with magnetic neutron diffraction unveiled ferromagnetic (FM) and antiferromagnetic (AF) phases below a temperature of ≈ 12 K depicted in Figs. 1 ▸ and 2 ▸ (Martinelli *et al.*, 2023 ▸). Both phases contribute axial and polar magnetic multipoles to our diffraction patterns. They include rotation of the sample about the reflection vector (an azimuthal angle scan).

Axial dipoles represent atomic magnetic moments in Figs. 1 ▸ and 2 ▸. Bragg spots in the FM phase satisfy reflection conditions for the parent structure. In consequence, Thomson scattering contributes to diffraction amplitudes in which the orientation of the photon polarization is unchanged, namely, (σ′σ) and (π′π). It is absent in the amplitude for rotated polarization (π′σ) equation (4), which features an axial dipole and Templeton–Templeton scattering (Templeton & Templeton, 1985 ▸). Equation (4) is correct for X-ray diffraction enhanced by an electric dipole–electric dipole (*E*1–*E*1) absorption event. A corresponding result for the electric quadrupole-electric quadrupole (*E*2–*E*2) absorption event is available from the electronic structure factor equation (3) and universal expressions for all diffraction amplitudes (Scagnoli *et al.*, 2009 ▸). Dirac quadrupoles and octupoles (polar magnetic multipoles) are revealed in by the parity-odd *E*1–*E*2 absorption event. The corresponding amplitudes equations (5) and (6) produce space-group forbidden Bragg spots. Likewise, all Bragg spots in the AF phase. In this phase, *E*1–*E*1 amplitudes (π′σ) and (π′π) in equations (8) and (9) contain axial dipoles alone. An anapole contributes to the *E*1–*E*2 amplitude (σ′σ) equation (10), whereas for the same reflection condition and an *E*1–*E*1 absorption event (σ′σ) = 0.
